# Neuropeptides, New Ligands of SARS-CoV-2 Nucleoprotein, a Potential Link between Replication, Inflammation and Neurotransmission

**DOI:** 10.3390/molecules27228094

**Published:** 2022-11-21

**Authors:** Julien Henri, Laetitia Minder, Kevin Mohanasundaram, Sébastien Dilly, Anne Goupil-Lamy, Carmelo Di Primo, Anny Slama Schwok

**Affiliations:** 1 Laboratoire de Biologie Computationnelle et Quantitative, Institut de Biologie Paris-Seine, UMR-CNRS 7238, Sorbonne Université, F-75005 Paris, France; 2 Institut Européen de Chimie et Biologie (IECB), CNRS, INSERM UAR 3033, US001, Univ. Bordeaux, F-33000 Bordeaux, France; 3 Saint Antoine Hospital, Centre de Recherche Saint Antoine, Sorbonne Université, Biology and Cancer Therapeutics, INSERM U938, F-75231 Paris, France; 4 Biovia, Dassault Systèmes, 10 Rue Marcel Dassault, CS40501, CEDEX, F-78946 Vélizy-Villacoublay, France; 5 CNRS, INSERM, ARNA, UMR 5320, U1212, IECB, Univ. Bordeaux, F-33000 Bordeaux, France

**Keywords:** nucleocapsid or nucleoprotein, SARS-CoV-2, structure-based drug discovery, alphafold model and molecular dynamics, neuroinflammation, brain fog, neuropeptides, neurotransmission, metabolism

## Abstract

This work identifies new ligands of the nucleoprotein N of SARS-CoV-2 by in silico screening, which used a new model of N, built from an Alphafold model refined by molecular dynamic simulations. The ligands were neuropeptides, such as substance P (1-7) and enkephalin, bound at a large site of the C-terminal or associated with the N-terminal β−sheet. The BA4 and BA5 Omicron variants of N also exhibited a large site as in wt N, and an increased flexibility of the BA5 variant, enabling substance P binding. The binding sites of some ligands deduced from modeling in wt N were assessed by mutation studies in surface plasmon resonance experiments. Dynamic light scattering showed that the ligands impeded RNA binding to N, which likely inhibited replication. We suggest that the physiological role of these neuropeptides in neurotransmission, pain and vasodilation for cholecystokinin and substance P could be altered by binding to N. We speculate that N may link between viral replication and multiple pathways leading to long COVID-19 symptoms. Therefore, N may constitute a “danger hub” that needs to be inhibited, even at high cost for the host. Antivirals targeted to N may therefore reduce the risk of brain fog and stroke, and improve patients’ health.

## 1. Introduction

The nucleoprotein N is one of the four structural proteins of SARS-CoV-2 virus [1,2,3,4]. N is present in large number of copies. As such, N is one of the major targets for antibody development and has been widely used for COVID-19 detection in the present pandemic outbreak. N binds the long viral RNA genome and is associated in the ribonucleoprotein (RNP) complex required for viral replication [5,6]. N is also involved in the formation of new virions through its interactions with the membrane protein M. Besides these functions, N acts as a mediator of inflammation. N represses the host antiviral response (as RNA interference and RIG-I mediated interferon) [7,8]. N targets the stress granule protein G3BP1, an essential antiviral protein known to induce innate immune response [9]. Importantly, N is associated with long-term SARS-CoV-2-specific immune and inflammatory responses, since the frequency of N-specific interferon-γ-producing CD8^+^ T cells decline more rapidly in long-hauler COVID-19 patients [10]. N also activates endothelial cells dysfunction, leading to vasculopathy and coagulopathy observed in some COVID-19 patients [11]. N is thus an important target for the development of antivirals [12].

It is recognized that SARS-CoV-2 induces an early host inflammatory response that activates a cyclooxygenase-2 (COX-2) inflammatory cascade associated with NF-κB activation [13,14]. Indeed, in a mouse model, N protein promoted the expression of pro-inflammatory cytokines and triggered lung injury via NF-κB activation [11,15]. The use of anti-inflammatory drugs, especially non-steroidal anti-inflammatory drugs (NSAIDs), in the initial outpatient stage of COVID-19 appears to be a valuable therapeutic strategy [16]. We identified, using a structure-based approach a cyclooxygenase (COX) inhibitor, naproxen, as an antiviral against SARS-CoV-2 and Influenza A virus that combines anti-inflammatory properties [17,18,19,20]. We showed that naproxen bound to SARS-CoV-2 N-terminal domain (NTD) in vitro and reduced the viral load of infected cells. Naproxen protected lung cells against viral injury in a model of lung epithelium, in contrast with celecoxib or paracetamol [17]. NTD was also shown to bind AMP, NADPH and single-stranded RNA [3,12].

In this paper, we extend our previous work and identify by in silico screening new ligands of the full-length N protein (FL). We report that the nucleocapsid can sequester in a large cavity of its C-terminal various host neuropeptides involved in neurotransmission, vasodilation, inflammation and ligands perturbing cell metabolism. This capture, although likely decreasing viral replication, may contribute to perturbations in brain function and metabolism. We discuss these hypotheses in light of the recent literature. Our study sheds light on the need to block N by antivirals which may overcome some long COVID-19 symptoms [21,22].

## 2. Results

### 2.1. Generation of Models of Full-Length Nucleoprotein N

**Figure 1:**
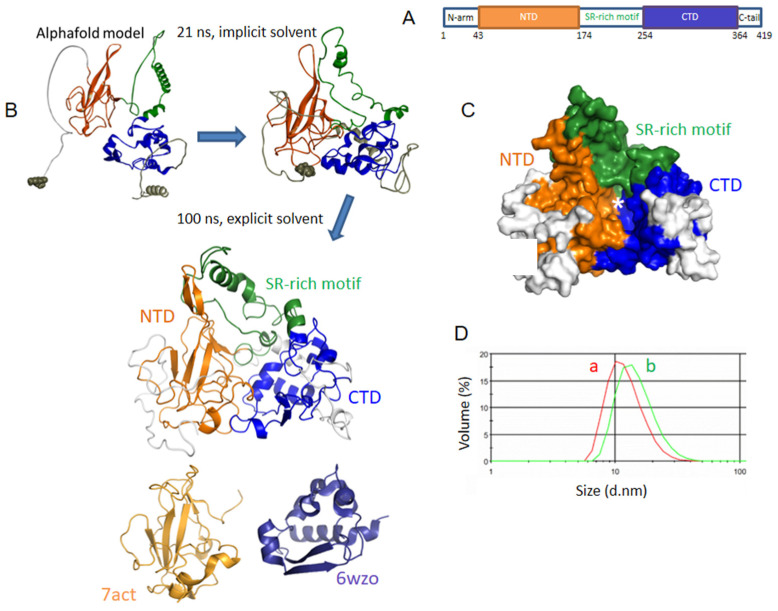
Sequence and model of the SARS-CoV-2 nucleoprotein wt N. (**A**) Schematic sequence of N, containing two structured domains at the N-terminal and C-terminal, NTD (brown) and CTD (blue), respectively. (**B**) Model of N, based on Alphafold, refined with MD simulations in implicit solvent, then in explicit solvent: the NTD and the RMN structure of the NTD (PDB 7ACT) [3] just below are shown in brown, and the CTD and the X-ray structure of the CTD (PDB 6WZO) just below are shown in blue, the SR-rich motif is presented in green, the other linkers in grey; the RMSD of the model compared to 7ACT is 4.7 Å and with one unit of the dimer of 6VYO [30] is 6.2 Å, the RMSD of the model with 6WZO [2] and 7DE1 [24] are 4.0 and 5.1 Å, respectively; the superimposition of the model and the experimental structures is shown in Supplementary Figure S1; (**C**) FL is shown as a surface with each domain colored with the same color code as depicted in A; note the large, mainly hydrophobic cavity, highlighted by the white star, at the C-terminal; see also Figure 2 for a visualization of the sites detected by Discovery Studio; (**D**) size distribution of the N protein (2 µM) in 20 mM Tris buffer pH = 7.9 containing 100 mM NaCl, (a) N alone and (b) N in the presence of RNA (TAR-polyA 6 µM) determined by dynamic light scattering (DLS).

Recent structures of both N amino-terminal domain (NTD) and C-terminal domain (CTD) have been solved by X-ray crystallography, Nuclear Magnetic Resonance (NMR) and electron microscopy [2,3,23,24,25]. N also bears dynamic, disordered domains: an SR-rich domain between the NTD and CTD, as well as N- and C-terminal tails (Figure 1A). The isolated NTD formed a monomer which bound RNA and other single-stranded nucleic acid fragments [2].

The N NTD structure presented a central antiparallel sheet of 3–5 β-strands (“the hand palm”) with a characteristic extrusion loop (“basic finger”) that can close on RNA upon binding [2,3]. The CTD also bound RNA and forms oligomers, mostly dimers [2]. The N protein also underwent liquid–liquid phase separation when mixed with RNA [1]. The full length (FL) N was reported to be a tetramer by size exclusion chromatography coupled to light scattering [2], but may also form a mixture of monomer and dimer [26]. However, the structure of the full-length N protein of SARS-CoV-2 is not yet available, partly because this recombinant protein is resistant to RNase treatments [26].

We present here a model of the full-length N protein initially generated by Alphafold and further optimized by MD simulations as schematized in Figure 1B, and described in the experimental section [27]. Figure 1B also shows the comparison of our model with the NMR structure of the NTD (PDB 7ACT [2]) shown in brown and the X-ray structure of the CTD (PDB 6WZO [3]) shown in blue, with root mean square deviations (RMSD) of 4.0 and 4.7 Å, respectively (see also Supplementary Figure S1 for the superimposition of the structures and the RMSD of two dynamic trajectories). Figure 1C shows that the protein presented a large cavity at its C-terminal domain, highlighted by a star, which could bind large ligands.

**Figure 2:**
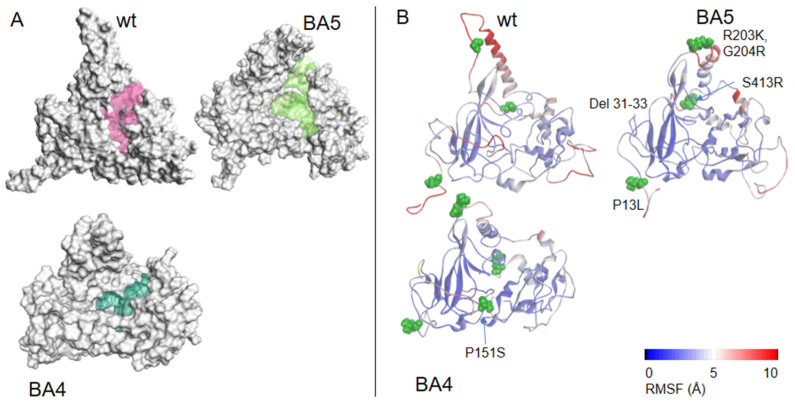
Models of wt N and variants. (**A**) Comparison of the modeled structures of N-FL shown as solvent-accessible surfaces: wt (Wuhan sequence); Omicron BA4 variant; Omicron BA5 variant. The C-terminal binding cavities are highlighted in colors. (**B**) 3D representation of the RMSF, as a measure of the protein flexibility of the main chain atoms (see also Supplementary Figure S1 for a 2D representation of the RMSF). The position of the deletion of the 31–33 residues is highlighted, the other mutations are shown in CPK. The RMSF follows a color code from blue (less flexible) to red (most flexible), note, in wt, BA4 and BA5 variants, the modifications of the fluctuations of the α-helix close to the R203K, S204R mutations located in close vicinity to the basic finger. These data highlight short- and long-range effects of the mutations on the protein flexibility, with a large enhancement in BA5 only missing the P151S mutation as compared to BA4 that may increase replication in the former variant.

While the NTD presented a number of suitable sites for ligands binding, these sites were small compared to the large cavity found at the C-terminal (Figure 2A), also detected in the isolated CTD X-ray structure [4,28]. Moreover, as this model of N was obtained based on the initial Wuhan strain of SARS-CoV-2 (wt FL), we also built the models of two Omicron variants of N to make sure that the detected cavity in wt FL would also bind ligands in more recent variants of N. Of the two Omicron variants of N, BA4 carried five mutations—P13L, P151S, R203K, G204R, S413R—and a deletion—31-33del—and BA5 had the same modifications but no mutation in P151 [29]. Figure 2A shows modifications of the cavities in the two variants, which only differed by the mutation of one residue. The latter cavity in BA5 was shifted apart from the β-sheet. The RMSD of the main chain atoms with respect to the wt ones were 1.57 Å and 1.28 Å in BA4 and BA5, respectively.

Associated with these changes in the cavity, we measured the protein flexibility of the three proteins, estimated by the root mean square fluctuations (RMSF). The model of wt N FL suggested that the α-helix close to the basic finger in the NTD was quite mobile (when the protein was not engaged in interaction with ligands), with flexibility of some linkers as part of the serine-rich domain and the C-tail (see also Supplementary Figure S1).

The R203K, G204R mutations in both variants tilted the edge of the basic finger that binds RNA towards the linker that carries these two positively charged mutated residues. Interestingly, the flexibility of the α-helix edge (R209) in close proximity to the R203K, G204R mutations was enhanced in the BA5 variant as compared to BA4, which may help to grasp the RNA more quickly and/or more efficiently. The data suggested subtle, long-range effects of the mutations (here the mutation status of residue 151 influencing the mobility of R209) that may modify replication. The N variant carrying the R203K, G204R mutations increased the infectivity, fitness and virulence of SARS-CoV-2 [30]. Altogether, the variants presented a modified cavity as compared to wt N, which nevertheless remained quite large and very flexible.

### 2.2. Characterization of Recombinant N, N-NTD and Its Mutants and N Full Length N-FL

The recombinant NTD protein was expressed as previously described [17]. NTD purified as a monomer deduced from SEC-MALS analysis [4]. The full-length protein (FL) was expressed and purified according to a similar protocol than that for NTD. However, the purified FL found in the soluble fraction remained contaminated by bacterial nucleic acids as attested by an absorbance ratio 260 nm/280 nm ranging between 0.9 to 1.3, despite the use of benzonase and RNase [26]. Instead, we used the insoluble fraction that was denatured with 6 M urea and then renatured in Tris-NaCl buffer. In these conditions, the absorbance ratio 260 nm/280 nm decreased to 0.47, consistent with no significant contamination by bacterial nucleic acids. The FL was then tested by DLS for its ability to bind RNA (TAR-polyA). The size of the native protein with bacterial nucleic acids contamination was 14.5 ± 1.0 nm in volume (data not shown). As shown in Figure 1D, the size of the protein depleted from nucleic acids contaminants was 12.0 ± 0.4 nm in volume (14.0 ± 0.3 nm in intensity) that increased to 15.1 ± 0.3 nm (20.3 ± 0.3 nm in intensity) upon addition of RNA (Figure 1D), showing that the protein is functional. By comparison, the size of the NTD monomeric protein was 6.0 ± 0.3 nm and increased to 15.7 ± 0.5 nm in intensity in the presence of nucleic acids. The size of NTD could not be fitted by a spherical model to obtain its value in volume. To further characterize FL protein, the apparent melting temperature (Tm) of the protein alone was determined by DLS as a function of temperature. FL presented a first transition at 43 ± 1 °C followed by a denaturation at about 54 °C, (Supplementary Figure S2), consistent with the hypothesis that the protein could be (at least partly) dimeric at 25 °C, with a transition to random coil upon heating at 54 °C, a transition also determined by circular dichroism [31]. Preliminary CD experiments showed that the spectrum of FL is dependent upon the protein concentration as previously reported.

### 2.3. Virtual Drug Screening on NTD and FL

We performed virtual screening to identify ligands binding to the large C-terminal cavity of the full-length protein (Figure 2A) using the data base of the Sigma-Aldrich catalog. We also screened for ligands binding to NTD. We first used the Libdock software of Discovery studio. Then, docking of the interesting hits was further repeated using CDocker. Finally, MD simulations were performed on the best hits. Table 1 and Table 2 summarize the mean-full hits both in terms of docking and biological function.

- *Ligands involved in metabolism*

Dihydrofolate (DHF) and tetrahydrofolate (THF) are, respectively, the substrate and product of the enzyme dihydrofolate reductase (DHFR).

**Figure 3:**
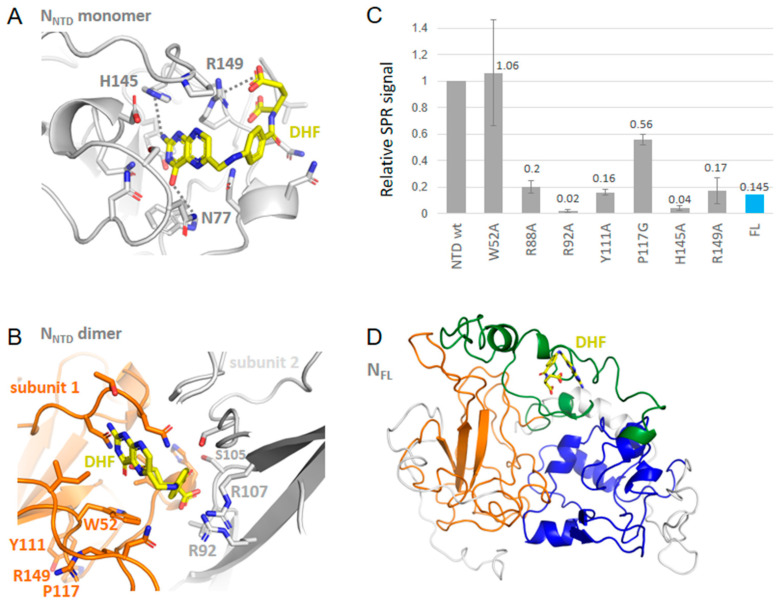
Interactions of N with dihydrofolate DHF. (**A**) Complex of monomeric NTD with DHF shown in yellow based on PDB 7ACT; (**B**) Complex of dimeric NTD with DHF based on PDB 6WYO; note the presence of cation–π interactions in both complexes; (**C**) SPR signal of wt NTD or mutants or of wt FL with DHF (10 µM) in 20 mM HEPES buffer: note the large decrease of the signal with the H145A and R149A mutants as predicted in A; the decrease with the R88A, R92A, and Y111A mutants with a small decrease in the P117G mutant, are in agreement with the structure in (**B**); (**D**) shows the modeled complex of DHF with FL, also observed in (**C**).

DHF binding to NTD: DHF interacted with NTD in its monomeric and dimeric forms (Figure 3A,B, respectively). It was anchored by arginine R149 (or R107 in NTD dimer) forming a π-cation complex with one of the aromatic rings of DHF, a π−π complex with H145 and further stabilized by a number of H bonds or electrostatic interactions with G44, N77, N150 (NTD monomer) and R92, S105 (NTD dimer). DHF binding on one face of the protein induced a compaction of the basic finger binding RNA on the other face, suggesting long range or allosteric effects. This compaction involved, in particular, R88 stabilized by salt bridges with D98 and E118. As R88, together with R92 and R107, have been all involved in RNA binding to NTD by NMR and further confirmed by mutation studies [2], it is likely that DHF binding strongly reduced RNA binding to N NTD.

DHF in FL (Table 2) Although DHF formed stronger polar interactions in FL than DHF in NTD, it did not form multiple hydrophobic and π–π interactions as in NTD.

AICAR (Table 1) AICAR is an agonist of AMPK; it bound through electrostatic interactions at a site at the basic finger where RNA binds, involving K61, R89, R93, R95, K102 and R107 via its charged and polar groups.

Lauroyl-CoA in FL (Table 1 and Table 2) The long aliphatic chain of lauroyl CoA was recognized by hydrophobic interactions with a series of five leucines and isoleucines residues and H356, F286 while its polar moiety forms H-bonds with S187 and S188 and K261 and R259 residues.

- *Ligands involved in the COX-prostaglandins pathway:*

The NSAIDs as naproxen bound the NTD [18]. They also can bind the C-terminal cavity although their size was not fitted to the large cavity. Table 2 describes the binding of acetamine at the C-terminal. Additionally, prostaglandins A2 and F2 and other eicosanoids bound FL with low affinity, further indicating the importance of this pathway in the host response to viral infection.

- *Neuropeptides:*

**Figure 4:**
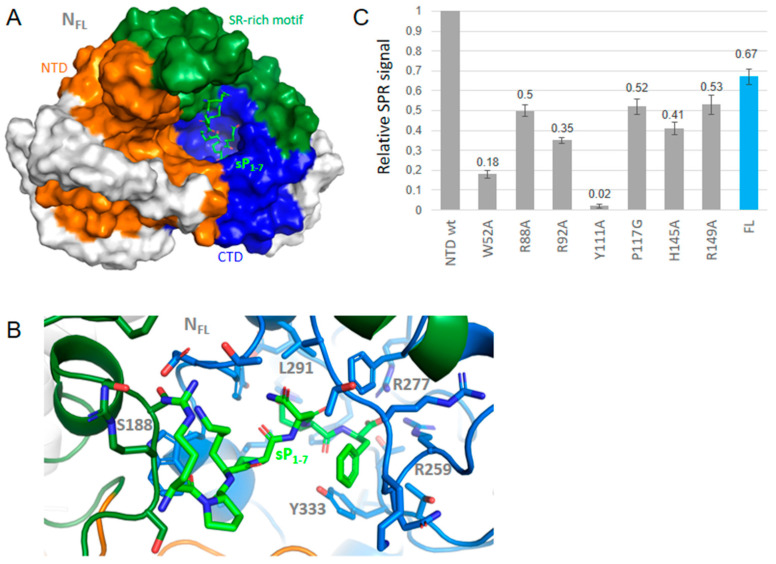
Interactions of wt N with substance P (1-7). (**A**) shows that the binding site of substance P (1-7) in a large binding site at FL C-terminal; (**B**) zoom on the complex of N with substance P (1-7) shown in green that formed multiple stacking and hydrophobic interactions, further stabilized by electrostatic and polar interactions; (**C**) consequently, the effect of the NTD mutations on the SPR signal was mainly observed with Y111A and W52A, with little effect seen on the other mutations. Substance P (1-7) also bound the N FL protein as measured by SPR.

**Figure 5:**
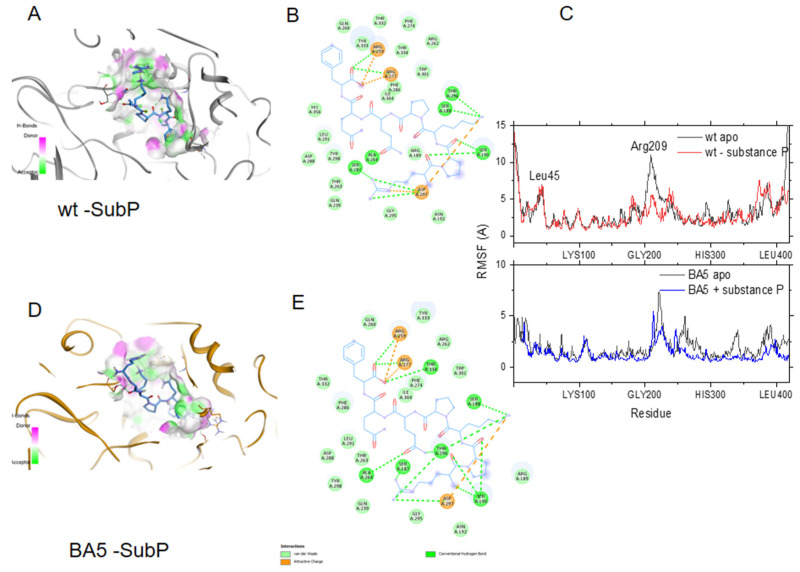
Binding of substance P (1-7) to the wt and the BA5 variant of N. (**A**) Binding site of substance P (1-7) in the wt N colored according to H-bond donor (pink) or acceptor (green) of the cavity; (**B**) 2D plots of the interactions of substance P (1-7) with wt N. Hydrogen bond and ionic interactions are shown as dashed green and orange colored lines, respectively; (**C**) upper: comparison of the flexibility of the wt apo protein (black line) with the wt-substance P (1-7) complex (red line); lower: comparison of the RMSF of BA5 apo (black line) and BA5- substance P (1-7) (complex (blue line); note the large decrease in the movement of the α-helix (R209) due to peptide binding in both complexes; (**D**) Binding site of substance P (1-7) in BA5; (**E**) 2D plots of the interactions of substance P (1-7) with BA5.

**Figure 6:**
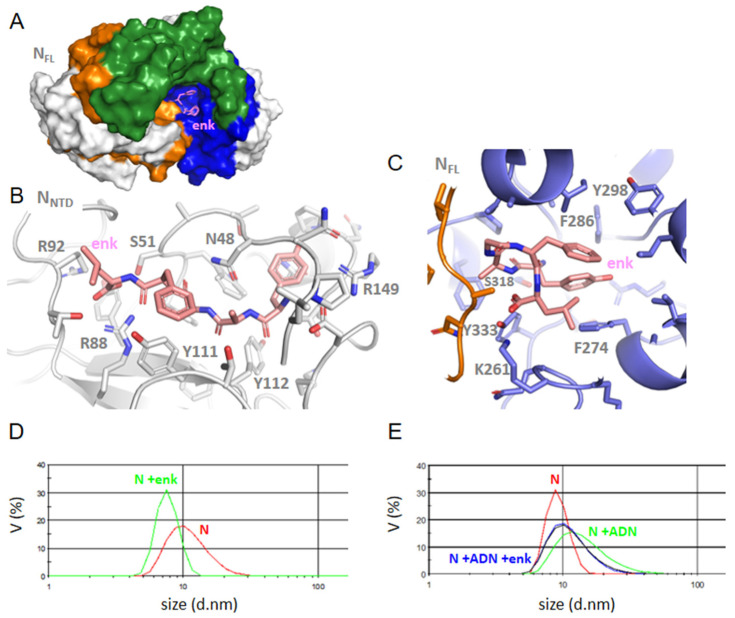
Interactions of Leu-enkephalin with NTD or FL. (**A**) enkephalin binding in CTD site in N FLwt, (**B**) the zoom of NTD-enkephalin complex, (**C**) FL-enkephalin complex; note the multiple hydrophobic interactions of this ligand in FL with P274, F286, Y298 and Y333, while the aromatic residues of the NTD β-sheet are involved in ligand binding in the NTD; (**D**) size distribution of N and its complex with enkephalin, which decreased its size as compared to the size of N alone; (**E**) the size of the N-DNA complex was reduced upon addition of enkephalin, in agreement with a competition of the ligand with nucleic acid binding to N FL.

Substance P (1-7) in FL bound at a similar site than observed with other neuropeptides, enkephalin, cholescystokinin and lauroyl coenzyme A (Figure 4, Figure 5 and Figure 6 and Table 2). Substance P (1-7) formed marked hydrophobic interactions with F286, Y298, I304 and Y333 as observed for enkephalin, and also binds S188 and R189 (Figure 4). This binding also resulted in a compaction of the RNA-binding finger, stabilized through interactions involving R95, D98, K100 and T205 although no direct interaction with the NTD was observed, including long-range effects between different sub-domains of the protein. Other fragments of substance P (4–11) also bound FL at a similar site and interaction energy than the P (1-7) fragment.

To further test whether substance P (1-7) may bind the BA4 and BA5 variants of N, we introduced the relevant mutations and deletion in the wt FL-substance P (1-7) model. Figure 5 shows that substance P (1-7) tightly bound to both wt and BA5 variant of N with similar interactions. Figure 5C also highlights the decrease of flexibility of N due to peptide binding in the structural element involved in RNA binding, in agreement with the competition between the ligand and RNA for binding to N (see below Table 3).

Enkephalin in NTD (Figure 6): Enkephalin bound at a close-by site from that of DHF via electrostatic interactions with R88 and R92. In addition, enkephalin extended toward the central β−sheet forming hydrophobic and polar interactions with Y111 and Y112. This binding induced some structuration of the linker carrying H145 and R149 in a short α−helix.

Enkephalin in FL (Figure 6A,C): Enkephalin interacted with FL through hydrophobic and π–π interactions with F274, F286, F298 and Y333 surrounding the ligand, stabilized by K261 and S318. Even though enkephalin bound FL through multiple hydrophobic interactions in the C-terminal cavity, it “closed” the basic finger on the N-terminal by H-bonds involving R95 with G97, G99 and T205, again suggesting long-range effects within the N structure. This hypothesis is confirmed by competition studies of enkephalin with RNA binding to N monitored by DLS (Paragraph 2–5).

Although we did not test directly the binding of enkephalin in the BA4 and BA5 variants of N FL, we anticipate that enkephalin would accommodate even more easily the variants than substance P as it can adopt an extended or folded conformation.

Cholescystokinin similarly bound FL through extensive hydrophobic interactions in the C-terminal cavity and also extended towards the N-terminal β-sheet via R107 and Y109. This binding mode was similar to that observed for substance P (Table 2).

- Hemin in FL:

Hemin bound FL through hydrophobic interactions with V270, L291, I304, I337 and π-π stacking with F274, in agreement with [32]. The stability of the complex was lower than that observed with the neuro/vasopeptides (Table 2).

### 2.4. SPR Study of DHF and Substance P (1-7) Interactions with NTD WT and Mutants, and FL

To comparatively assess the binding of DHF and substance P (1-7) to N, we generated mutants of the NTD recombinant protein by targeting some of the residues involved in ligand binding as detailed in experimental Section 4.1.3. The effect of such mutations on DHF binding to NTD are shown in Figure 3. Mutations H145A and R149A strongly reduced the signal of DHF as compared to DHF binding to NTD (monomer) wt as expected from the modeling. In addition to these mutations, we also observed strong effects of the mutations R92A, Y111A and R88A. These residues corresponded to those involved in DHF binding in NTD dimer. As compared to DHF signal in NTD, binding of DHF to the full-length protein was smaller. The relative signal ratio (DHF in NTD)/(DHF in FL) was dependent on the buffer used. It is possible that the oligomeric status of FL was different in HEPES buffer supplemented with 0.05% Tween, compared to Tris buffer. FL was reported to be either tetrameric or a mixture of monomer and dimer [2,26]. Monomer or dimer form of N FL, in particular in the C-terminal provided more accessible site(s) for ligand binding than the tetrameric form did. DHF K_D_ for binding to NTD was about 1 µM, deduced from the variation of the signal as a function of DHF concentration in the range 0.3–10.0 µM.

Substance P (1-7) binding to NTD and FL was also observed as shown in Figure 4C. This large ligand bound NTD and exhibited relatively small effects of the mutations with notable exceptions of W52A and Y111A. This suggested that substance P (1-7) bound on the β-sheet at the conserved sequence of the five aromatic residues W^108^YFYY^112^. Substance P (1-7) binding to N therefore seemed driven by hydrophobic interactions, consistent with the sequence of the peptide. It was also in line with both the binding of the peptide to FL seen by SPR and the multiple hydrophobic contacts of this peptide in the C-terminal cavity of the FL protein suggested by modeling.

### 2.5. FL Interactions with the Ligands by DLS; Competition with Single-Stranded DNA or RNA Binding

To further address whether the ligands may modify the oligomeric status of the FL protein, we performed dynamic light scattering experiments. FL (2 µM) presented a main peak at 12.0 ± 0.6 nm in volume (14 nm in intensity); the size of the complex decreased to 10.3 and 7.8 ± 0.3 nm upon addition of 2 µM and 6 µM enkephalin, respectively (Figure 6D). As shown in Table 3, the same trend was observed upon addition of naproxen, indomethacin, substance P (1-7) or a porphyrin ZnTPPS with a larger decrease observed with substance P (1-7), ZnTPPS and indomethacin. This suggested that ligand binding decreased the oligomerization state of the protein. We then tested whether ligand binding to FL could be competitive with nucleic acids binding. Table 3 indeed shows that the substance P (1-7) or enkephalin (Figure 6E) are competitive with nucleic acids binding to FL as the size of the RNA-FL or DNA-FL complex about 14 nm observed without ligand always decreased by addition of the ligands.

### 2.6. FL Interactions with ZnTPPS

**Figure 7:**
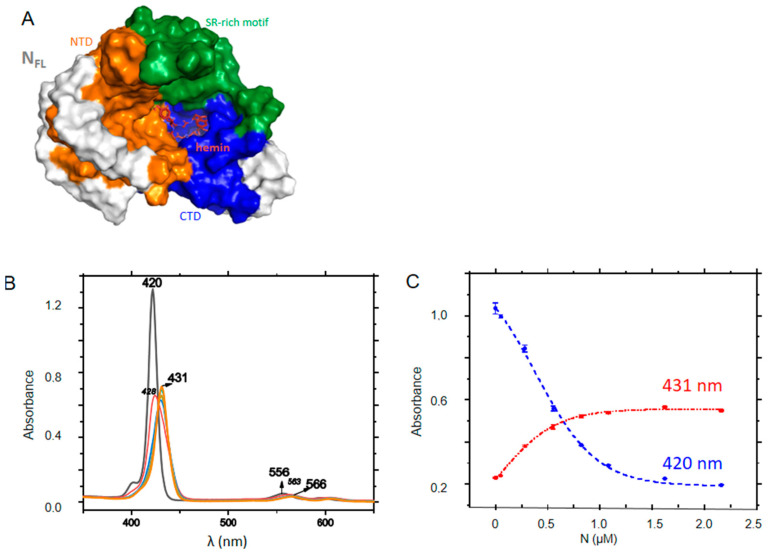
Interactions of FL with ZnTPPS. (**A**) the porphyrin shown in red binds at the C-terminal cavity of FL (See also Table 2). (**B**) Absorption changes of ZnTPPS upon titration with FL; (**C**) Absorbance at Soret maxima of the free (420 nm) or FL-bound (431 nm) porphyrin; the dashed lines correspond to fits of the experimental data according to a dose-response function, yielding K_D_ = 0.40 ± 0.10 µM.

In our in silico screening, we identified hemin as a potential ligand binding to FL C-terminal site as recently proposed; hemin binding to N was shown to reduce viral replication [32]. Figure 7A shows that the binding site of hemin colored in red. Here, we chose a water-soluble, negatively charged porphyrin with properties of a photosensitizer, able to produce singlet oxygen and ROS in the perspective of a potential antiviral which could sensitize infected tissues containing N. ZnTPPS has a Soret band at 420 nm and two Q bands, being a metalated porphyrin. Upon addition of FL, the Soret band decreased and red-shifted to 431 nm with the presence of an isosbestic point at 426 nm, while the first Q band shifted from 557 to 561 nm. The data were repeated at various concentrations of ZnTPPS, yielding a K_D_ = 0.40 ± 0.10 µM (Figure 7B,C).

## 3. Discussion

In this work, we identified new ligands of N in the perspective of drug repurposing, with perspectives in basic understanding of N functions.

The model of FL, the full-length nucleoprotein of the wt sequence we built, was generated by Alphafold, with subsequent refinements using molecular dynamics simulations. The model clearly showed that the protein is very flexible, nevertheless the simulations successfully produced a folded model, presenting a large cavity at the C-terminal of the protein able to bind ligands with a broad distribution of molecular weights, similar to the cavity found in the X-ray structure of the isolated C-terminal protein. By introducing the mutations and deletion found in the Omicron BA4 and BA5 variants of N, we also detected a large cavity at the C-terminal, although modified as compared to the wt one. It is interesting to note the increased flexibility of the BA5 variant as compared to that of the BA4 variant, in particular in a linker carrying the 203–204 mutations just opposite the basic finger that bound RNA. This suggested a better fitness to replication in the BA5 variant because of a better/faster adaptation to the viral RNA as compared to that of the BA4 variant [30].

The ligands we identified by in silico screening are likely to decrease viral replication as: (i) the ligands competed with RNA binding; (ii) the ligands reduced FL (oligomer) size. Since N is known to oligomerize upon binding to RNA, both mechanisms are expected to interfere with viral replication. Four ligands we identified in our screening were indeed recently shown to decrease viral replication: naproxen, indomethacin, DHF and hemin [17,18,34,35]. The DLS data were consistent with a competition of DHF, naproxen, indomethacin, enkephalin, substance P (1-7) and ZnTPPS with RNA binding to FL.

Besides its roles in viral transcription and replication, N is involved in immunity via its control in host interferon release, in cytoskeleton rearrangement. The literature further suggests additional roles of N that our data may further highlight, as detailed below.

### 3.1. N as a Mediator of Inflammation and Its Effect in Long-Haul COVID-19

A sustained inflammation that extended well beyond clearance of the primary infection was observed in long-hauler patients and also found in a hamster model of SARS-CoV-2 infection [15,36]. The expression of prostaglandin receptors was increased in patients with severe COVID-19, as part of the cytokine storm developed by these patients [37]. Accordingly, our virtual screening identified prostaglandin E2 and other eicosanoids as N FL binders, these metabolites belonging to the COX–arachidonic -eicosanoids (including prostaglandin) pathways of inflammation and metabolism as an (early) host response triggered by the viral infection, in agreement with recent reports [16,38]. Treatment of patients hospitalized for mild and moderate COVID-19 with the COX inhibitor indomethacin helped the patients to recover [39]. Naproxen inhibited replication and reduced inflammation in a model of reconstituted lung epithelium [17]. In silico studies suggested that naproxen and acetaminophen may not only bind the N protein but also SARS-CoV-2 main protease, the RBD domain of spike and the polymerase [40].

The cytokine storm could also be considered as a possible driving factor for the expansion of neuropathies after severe COVID-19 infection, contributing to the chronic pain that appeared after acute infection recovery. The cytokine storm was at least partly mediated by (lung-resident and brain-penetrating) macrophages blocked in an M1 state that released pro-inflammatory cytokines as IL6 [41,42,43]. The neuropeptides identified in this work were known to have a role in inflammation: substance P is a mediator of inflammation and pain [41,44,45], while enkephalin had the opposite role to release pain [46,47]. In dopaminergic neurons, subpicomolar levels of substance P activated NADPH Oxydase-2 (NOX2) to increase ROS concentrations and subsequently synergistically activated the MAPK and NF-κB pathways, contributing to a potentiated pro-inflammatory cytokine production [47,48]. In addition, substance P and cholestocystokinin were vasoactive peptides [47]. We hypothesize that the sequestration by N of the identified ligands of N could have consequences for the host in terms of endothelial dysfunction, micro-thrombus in the brain and in other microvessels, with increased risk of stroke. Indeed, in the brain of SARS-CoV-2 infected non-human primates, the occurrence of a neuronal injury, brain micro-hemorrhages and hypoxia and rare viruses in brain-associated endothelium where N co-localized with the von Willebrand factor were observed [21]. It was also proposed that substance P and bradykinin, were likely to drive microvascular permeability, and be responsible for a phenomenon called «vasoactive peptide storm» as part of the development of COVID-19 pathology [48]. An agonist of cholecystokinin A (CCK-A) showed benefit in reduction of inflammation, among those hospitalized with moderate COVID-19 [49].

Therefore, we propose the hypothesis that the identified neuropeptides (and/or their fragments) could contribute to long-term inflammation in the CNS via N and possibly other mechanisms.

### 3.2. N Implication in Immunity, with Possible Long-Term Neurological Effects and Putative Viral Latency

N implication in immunity of the host against SARS-CoV-2 was expressed at multiple levels.

(i) *T cells*: Patients with persistent symptoms over 4 months following COVID-19 onset presented a lower frequency of CD8^+^ T cells expressing CD107a, a marker of degranulation, in response to Nucleocapsid (N) peptide pool stimulation, and a more rapid decline in the frequency of N-specific interferon-γ-producing CD8^+^ T cells [10].
(ii) *Opioid peptides*: Immune system and neuronal system cross-talk; this signaling is mediated by various molecules such as opioid peptides such as enkephalin. Enkephalins can impact lymphocytes proliferation, antibody synthesis. Enkephalin can enhance the release of pro-inflammatory cytokines like IL6 [50]. The cross-talk can take place between cytokines as CCL2 and opioid peptides and alter nociceptive synaptic transmission [51]. Thus, more work is required to test whether the enkephalin peptide identified here as a ligand of N may increase pain and immune disorders in the context of COVID-19, despite its physiological pain release function.
(iii) *Vasoactive peptides*: Among the possible routes through which SARS-CoV-2 can invade the CNS, SARS-CoV-2 can directly invade the vagus nerve and retrograde into the CNS, or indirectly stimulate the enteric nervous system through immune pathways. Cholecystokinin could participate in this process. In addition, peripheral nerves may spread SARS-CoV-2 into the brain through the retro-neural route, including the olfactory nerve, trigeminal nerve, glossopharyngeal nerve and vagus nerve. Substance P is the main neuropeptide, neuromodulator and neuro-hormone of the trigeminal ganglion (TG), associated with nociception and inflammation through its receptor, the neurokinin-1. As observed with other viruses such as herpes or HIV, it was hypothesized that SARS-CoV-2 virus infection might become latent if it is acting through TG [41,45], involving substance P action.

### 3.3. N as a Mediator of Perturbed Metabolism via Its Ligands, with Possible Long-Term Effects

DHF and THF linked N to DHFR, an important enzyme that participates to DNA synthesis, being coupled to methionine metabolism. Additionally, folate exerted a protective role in the cardiovascular system [52,53]. Moreover, folate levels were usually low in patients with viral infection [54,55]. The reduction of viral replication by DHF/THF came at the expense of large perturbations of the host purine metabolism, nicely described in [35]; it is likely that the agonist of AMPK, AICAR would also have a dual effect on replication and lipid metabolism. The screening also identified Lauroyl-coA as a ligand of N, potentially linking N to beta oxidation of fatty acids and to triacylglycerol biosynthesis.

### 3.4. Zntpps as a Prototype of a Photoactive N Ligand for Photodynamic Therapy (PDT)

The binding site of hemin was deduced from docking [32], with a putative NLS signal (amino acids 258–268) at the edge of this site [33]. This site shared a number of predicted residues involved in the binding of the neuropeptides and DHF. Binding of ZnTPPS was demonstrated by spectroscopic changes of the porphyrin. ZnTPPS is one of the sensitizers that can release singlet oxygen and ROS upon light/ultrasound activation. Such compounds have been useful in the treatment of some cancers and proposed as antiviral treatment to inactivate SARS-CoV-2 [56].

### 3.5. Heme Sequestering by N and Its Potential Effect on NO and ROS Signaling

Dysfunctional, low hemoglobin levels, observed in patients with COVID-19 were possibly linked to well-characterized brain hypoxia [22,57]. Here, we further suggested that folate binding to N could also be responsible for an altered iron metabolism via perturbation in folate levels [35]. Moreover, we speculate that heme binding to FL could increase disruption of labile hemes from proteins such as guanylate cyclase, with potential effects in NO signaling, reduced oxygen/hypoxia sensing and/or impediment of their gaseous ligand binding such as O_2_, NO and CO, with all the known consequences of altered NO/O_2_ signaling in the cardiovascular system (eNOS), in neurotransmission, control of serotonin levels (nNOS) and in immunity (iNOS).

## 4. Materials and Methods

### 4.1. Models of N, in Silico Screening, Docking and Molecular Dynamics Simulations

#### 4.1.1. Models of wt N

The following X-ray structures extracted from the Protein Data Bank (PDB) have been used: N-terminal domain of SARS-CoV-2: 6VYO [58], 7ACT [2].

A computational model of the full-length nucleoprotein of SARS-CoV-2 was generated with the ColabFold [59] version of AlphaFold 2 [27] using an API hosted at the Södinglab based on the MMseqs2 server [60] for the multiple sequence alignment creation. 300 sequences were aligned on uniprot entry A0A6C0T6Z7 as query sequence and no template (419 residues). Predicted lDDT is highest in the NTD and CTD regions that were previously determined by experimental methods (PDB entries 7act, 6wzo).

MD simulations were performed based on Alphafold and initial minimization and equilibration steps for 21 ns in implicit solvent. The wt protein was prepared with Discovery Studio 2022 (Dassault Systèmes BIOVIA, Discovery Studio Modeling Environment, Release 2022, (San Diego, CA, USA): Dassault Systèmes 2022) the models were first protonated at pH 7.4, typed with CHARMM36 force field and the protein was placed in an orthorhombic box solvated with a pre-equilibrated solvent containing TIP3 waters, with a 15Å minimum distance from boundary. Two trajectories were generated with the NAMD 2.13 engine and CUDA acceleration as implemented in Discovery Studio 2022, by varying the Random Seed Number. The MD simulations were run for 100 ns each under constant pressure (NPT ensemble) at 300 K using a Langevin thermostat and piston, for temperature and pressure control, respectively, and long-range electrostatic interactions were computed using Particle Mesh Ewald (PME) algorithm [61]. The trajectory was analyzed with the Analyze Trajectory Protocol to compute the RMSD (Root Mean Square Deviation) and RMSF (Root Mean Square Fluctuation).

#### 4.1.2. Model of N Variants

This wt model was then used to generate the BA4 and BA5 variants of N with the MODELER algorithm [62] as implemented in Discovery Studio version 2022 (Dassault Systèmes BIOVIA, Discovery Studio Modeling Environment, Release 2022, San Diego: Dassault Systèmes 2022). For each of the two mutants BA4 and BA5, a 100 ns trajectory was also generated with the same protocol as described for the WT protein.

All the cavities were obtained with the Define Site from Receptor Cavities of Discovery Studio 2022 as shown in Figure 2.

#### 4.1.3. Ligand Screening and Docking to wt N

*In silico* screening of ligands that bind the protein structures was performed using the Sigma catalog data base and assessed in a two-step protocol. For this screening, we first used the docking program Libdock of Discovery Studio version 2021, with the exclusion of ligands with molecular weights above 2000 and according to the binding site(s) defined by cavity detection. A site-docking was then performed using Cdocker with energy minimization (Discovery Studio version 2021) to more precisely identify the ligand binding mode. For each protein, the most representative pose of the ligand was selected. The resulting protein–ligand complexes were finally refined by a molecular dynamics simulation using the CHARMm force field [63] and the standard dynamic cascade protocol of Discovery studio version 2021. This protocol started with a first minimization of 1000 steps using the Steepest Descent algorithm and a RMSD gradient of 1 Å, followed by a second minimization of 2000 steps using the Adopted Basis Newton–Raphson algorithm and an rmsd gradient of 0.1 Å. The third step involved heating from 50 K to 300 K, with a fourth step of equilibration during 1 ns and a fifth step, production. The time of the production step was initially set at 10 ns, but extension to 20 ns was applied if the ligand was not stable. Three replicas were carried out for each complex. For each trajectory, the displacement of the ligands was studied by RMSD calculation. The representative structure (i.e., with the smallest average rmsd from all other structures of the cluster) of the largest cluster of each complex was selected.

#### 4.1.4. Substance P (1-7) Bound to BA5 Variant

An homology model of BA5 in complex with substance P (1-7) was built with MODELER, using the wt-substance P (1-7) as a template. For each of the two mutants BA4 and BA5 in complex with the substance P (1-7), a 100 ns trajectory was also generated with the same protocol as described for the wt protein.

### 4.2. Chemicals and Oligonucleotides

We bought from Sigma Aldrich, St Quentin Fallavier, France: dihydrofolate, Leu-enkephalin, 5-Aminoimidazole-4-carboxamide ribonucleotide (AICAR), naproxen, indomethacin and Tris, NaCl, the oligonucleotides with or without TEG biotin tags were purchased with HPLC purification. The sequence of the 48 mer DNA was: 5′ATA TAT ATC TAT GTC CAT ATA TAT ATA AAA CAC AGC GTG TGT GTG TAA 3′. The sequence of TAR-polyA was: 5′ (A)_21_ GAA AGG AGC CUG GGA GCU CC 3′. Substance P (1-7) was synthesized by Genecust, Boynes, France. The plasmids for NTD, mutants and the full-length N were obtained from Genecust, Boynes, France.

### 4.3. Proteins Expression and Purification

The N-terminal domain of the nucleoprotein of SARS-CoV-2 (residues 50–173) with an N-terminal His6 tag was cloned in pET-28a vector (pET28a-His6-NP-NTD). Heterologous expression at 15 °C for 16 h in *E. coli* BL21 bacterial strain (DE3 (NEB, Evry, France). The recombinant protein (15 kDa) found in the soluble fraction was purified on a Ni^2+^-NTA affinity column and SP sepharose ion exchange chromatography, and presented a single band revealed by SDS PAGE. The yield was 1.5 mg of pure protein per liter of culture.

The expression of the FL protein was performed as described above. The insoluble fraction of the pellet was resuspended in lysis buffer containing 6 M urea for an hour at room temperature, the insoluble part was removed by centrifugation over 10 min. The purification involved an affinity Ni^2+^ column, with a first washing at 3 M urea, followed by washings in a 20 mM Tris-HCl pH = 7.9 and 1 M NaCl, and then increasing concentrations of imidazole (10, 20 and 50 mM). Elution was performed with 500 mM imidazole. Imidazole was then removed from the solution by dilution-ultrafiltration (centricon molecular weight cutoff 10,000, Millipore). The protein was then left at 4 °C for three days for renaturation in a 20 mM Tris-HCl buffer at pH = 7.9, 100 mM NaCl.

### 4.4. SPR Experiments

The recombinant FL protein was tagged with six His for purposes of identification and affinity purification, and the same principle was exploited for capture of the recombinant proteins on the sensor surface. Poly-histidine is a commonly used tag that can chelate with Ni^2+^ ions in complex with nitrilotriacetic acid (NTA, Biacore, Uppsala, Sweden), providing a convenient approach for capturing tagged constructs on Sensor Chip NTA. The FL protein was injected at a flow rate of 30 µL/minute; the ligand was injected after the protein capture; then, the protein or protein–ligand complex was removed from the surface by applying 350 mM EDTA, with a very good reproducibility. The experiments were usually performed in 20 mM HEPES buffer (Biacore), 100 mM NaCl and 0.05% Tween. The FL protein was captured at a concentration of 0.1 µM as the signal decreased at higher FL concentrations, presumably because the protein adopts higher oligomeric state(s). The NTD wt and mutant proteins were captured at a concentration of 1 µM for shorter times. In both FL and NTD proteins, signals of 5000 to 8000 RU were captured on the surface. The NTD proteins were always used as freshly prepared solutions. The ligands concentration range was usually in the range 0.3–10 µM.

### 4.5. Dynamic Light Scattering

The experiments were performed on a Malvern nanosizer apparatus. The temperature was set at 20 °C, and 10 scans with a duration of 10 s each were acquired in duplicate for each time and sample. The size distribution in the intensity of the scattered light was obtained using the Cumulants method from the instrumental software, yielding the hydrodynamic diameter. The N NTD concentration was in the range of 40–60 μM, the FL concentration was in the range of 1 to 10 µM in 20 mM Tris buffer at pH = 7.9 containing 100 mM NaCl. The melting experiments were performed at a heating rate of 1 °C/min over the range 30 °C to 65 °C in sealed disposable cuvettes. The apparent melting temperature, Tm, was determined by the first derivative of the melting curve.

## 5. Conclusions

The abundant N protein potentially could disrupt many signaling pathways, through sequestration of signaling molecules as the neuropeptides identified here or other useful metabolites at its large C-terminal cavity. An increased flexibility of N seemed to emerge in the BA5 N variant as compared to the BA4 one, which may both speed up replication and enhance ligand binding, with possible cooperative or long-range effects of the mutations/deletion. Although these neuropeptides usually signal in the pico-to nanomolar concentration range in physiological conditions, their concentrations in the blood, in the CNS and in TG can increase locally, in particular in COVID-19 patients [64,65,66,67,68,69,70], in line with the hypothesis suggested by this study. N may affect the cell metabolism via folate and AMPK and neurotransmission via neuro/vasoactive peptides. The physiological function of these ligands or their fragments would probably be altered consecutive to their association with N.

Altogether, the host likely senses the N protein as a “danger hub”; any possible mean seems to be undertaken to neutralize N. Therefore, instead of letting valuable neuropeptides be sequestered by N, blocking N with antivirals may decrease symptoms associated with long COVID-19, which accounts for 5–20% of the patients [22,71]. Based on the biological functions of the neuropeptides, we speculate that N could be involved in pain, neurological brain fog and inflammation and possibly immune imbalance and latency. Current NSAIDs as naproxen and indomethacin are readily available and bind to N; extended ligands could be also adequate to fill the C-terminal cavity of N. The hypothesis raised in this discussion offers a comprehensive picture that may link multiple long-haul COVID-19 symptoms through N, which is yet to be demonstrated by clinical data.

## Figures and Tables

**Table 1:** NTD and FL ligands identified by virtual screening and their reported biological functions.

NTD Ligands	FL Ligands	Biological Function
DHF	DHF	Metabolism
THF	THF	Metabolism
AICAR (AMPK agonist)	Lauroyl coA	Metabolism
	Hemin	Metabolism
Naproxen Acetamine Indomethacin	Prostaglandin E2 and F2 and *Other eicosanoids*	COX pathway/NSAID COX pathway/NSAID COX pathway/NSAID
	D Ala_2_-Leu_5_-Enkephalin YAGFL-OH (DADLE)	Neuropeptide involved in pain reduction, agonist of the μ− opioid receptor
	Substance P (1-7) RPKPQQF-OH Cholecystokinin DYMGWMDF (CCK8)	Neuropeptide mediator of inflammation, pain, and vasodilation, agonist of neuro- -kinin-1 receptor. Intestinal hormone peptide binding to a receptor on nerve fibers of the vagus nerve

**Table 2:** Summary of the ligand-N interactions obtained by modeling compared to the measured complex by surface plasmon resonance with wt or mutant NTD or wt FL.

Ligand		Protein	-CDocker Interaction Energy Kcal/mol	Critical Residues	Mutations SPR (% Inhibition/NTD wt)	K_D_
DHF	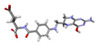	NTD monomer	58.0	G44, L45, N77, H145, R149, N150, N153	H145 (96%), R149 (83%)	1 µM
THF	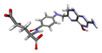	NTD monomer	61.7			
Naproxen	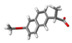	NTD monomer	38.0	W52, I146, R149 (site 1) [17] A50, R88, R92, R93 (site 2)	R88 (55%), R149 (44%)	4.4 ± 1.4 µM [17]
Acetamine	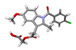	FL	39.7	F286, I304, S318, T334, G335, I337		
DHF		FL	57.9	R189, K233, K237, N239		
Enkephalin	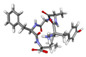	FL	64.4	A155, K261 ^a^, F274, F286, G287, Y298, I304, S318, Y333, G335, A336		
Lauroyl coA		FL	79.5	S187, S188, R189, K261 ^a^, R262 ^a^,		
Substance P (1-7)	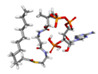	FL	82.4	S188, R189, R259 ^a^,T263 ^a^, A264 ^a^, R277, F286, L291, G295, Y298, Y333, T334, H356		
Cholecystokinin	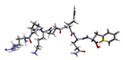	FL	82.0	A55, R107, Y109, V158, A264 ^a^, V270, L291, W301, P302, H356		
Hemin	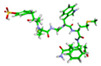	FL	47	A264 ^a^, V270,F274, L291, I304, A305, I337		
ZnTPPS	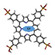	FL				0.4 ± 0.1 µM

^a^: Residue belonging to a putative nuclear localization signal NLS [32,33].

**Table 3:** Size of N and its complexes with the studied ligands with or without nucleic acids determined by DLS.

Ligand	Ligand Concentration (µM)	Size (nm) Intensity	Size (nm) Volume	N NTD/FL
none		5.3 ± 0.3 (80%)	Does not fit	NTD (60 µM)
none		13.2 ± 1.0 (85%)	12.0 ± 1.0 (96%)	FL 2 µM
Naproxen	2 6	12.2 ± 0.5 (40%) 8.7 ± 0.3 (60%)	11.3 ± 0.4 (99%) 8.9 ± 0.3 (96%)	FL 2 µM
Indomethacin	2 6	11.0 ± 0.4 (30%) 6.7 ± 0.3 (60%)	10.3 ± 0.5 (100%) 6.3 ± 0.4 (96%)	FL 2 µM
Enkephalin	2 6	12.6 ± 0.6 (31%) 8.7 ± 0.2 (40%)	11.3 ± 0.6 (96%) 8.0 ± 0.3 (100%)	FL 2 µM
Substance P	2 6	10.3 ± 0.6 (50%) 8.0 ± 1.0 (76%)	9.5 ± 0.6 (98%) 7.2 ± 1.1 (99%)	FL 2 µM
ZnTPPS	2	7.6 ± 0.6 (25%)	7.3 ± 0.6 (99%)	FL 2 µM
RNA (TAR-polyA)	4	21.5 ± 2.0 (75%)	15.1 ± 1.1 (98%)	FL 4 µM
48m-DNA	4	25.3 ± 3.1 (56%)	15.7 ± 1.2 (96%)	FL 4 µM
RNA + subP	4	16.1 ± 0.6 (75%)	11.3 ± 0.6 (97%)	FL 4 µM
DNA + subP	4	13.8 ± 0.6 (70%)	10.1 ± 0.6 (91%)	FL 4 µM
DNA + enkephalin	4	10.5 ± 1.2 (89%)	9.0 ± 1.7 (99%)	FL 4 µM

## Data Availability

Modeled structures can be available upon request to A.S.S.

## Supplementary Materials

The following supporting information can be downloaded at: https://www.mdpi.com/article/10.3390/molecules27228094/s1, Figure S1: Analysis of the models of N; Figure S2: Melting curve of recombinant FL determined by DLS.

## Sample Availability

Samples of the compounds are not available from the authors.

## Dedication

This work is dedicated to the so many long-hauler COVID-19 patients, with love and hope that this work will open new and useful insight to helping them.
