# Supplementary material for: Neuropeptides, New Ligands of SARS-CoV-2 Nucleoprotein, a Potential Link between Replication, Inflammation and Neurotransmission
Source: Molecules. 2022 Nov 21;27(22):8094. doi: 10.3390/molecules27228094 (PMC9698730; doi:10.3390/molecules27228094)

molecules

MDPI

Supplementary Materials

Neuropeptides, New Ligands of SARS-CoV-2 Nucleoprotein, a Potential Link between Replication, Inflammation and Neurotransmission

Julien Henri [1], Laetitia Minder [2], Kevin Mohanasundaram [3], Sébastien Dilly [3], Anne Goupil-Lamy [4], Carmelo Di Primo [5] and Anny Slama Schwok [3,*]

Supplementary Figure S1: Analysis of the models of N

A: sequence of N, with mention of the two structured domains, NTD and CTD, together with flexible linkers. The model is superimposed with the reported structures of the NTD and CTD, also shown in Figure 1A; B: Ramachandran plot of the wt model of N, the light and dark grey areas represent the highly preferred conformations ($\Delta \geq -2$) and the green points the highly preferred observations (90.75%), the black areas represent the preferred conformations ($-2 > \Delta \geq -4$) and the orange points preferred observations (7.8%), the grey grid represent the questionable conformations and the red points the questionable observations (1.44%) corresponding to five residues out of 419, namely: Ser37, Ala119, Leu121, Asn196, Ser232); C: RMSD (in Å) of two 100 ns MD trajectories in N FL wt and N Fl wt with substance P (1-7). D: Comparison of the 2D representation of the RMSF (in Å) of the wt (blue) and BA4 variant (green, upper graph) and of the wt and the BA5 variant (orange).

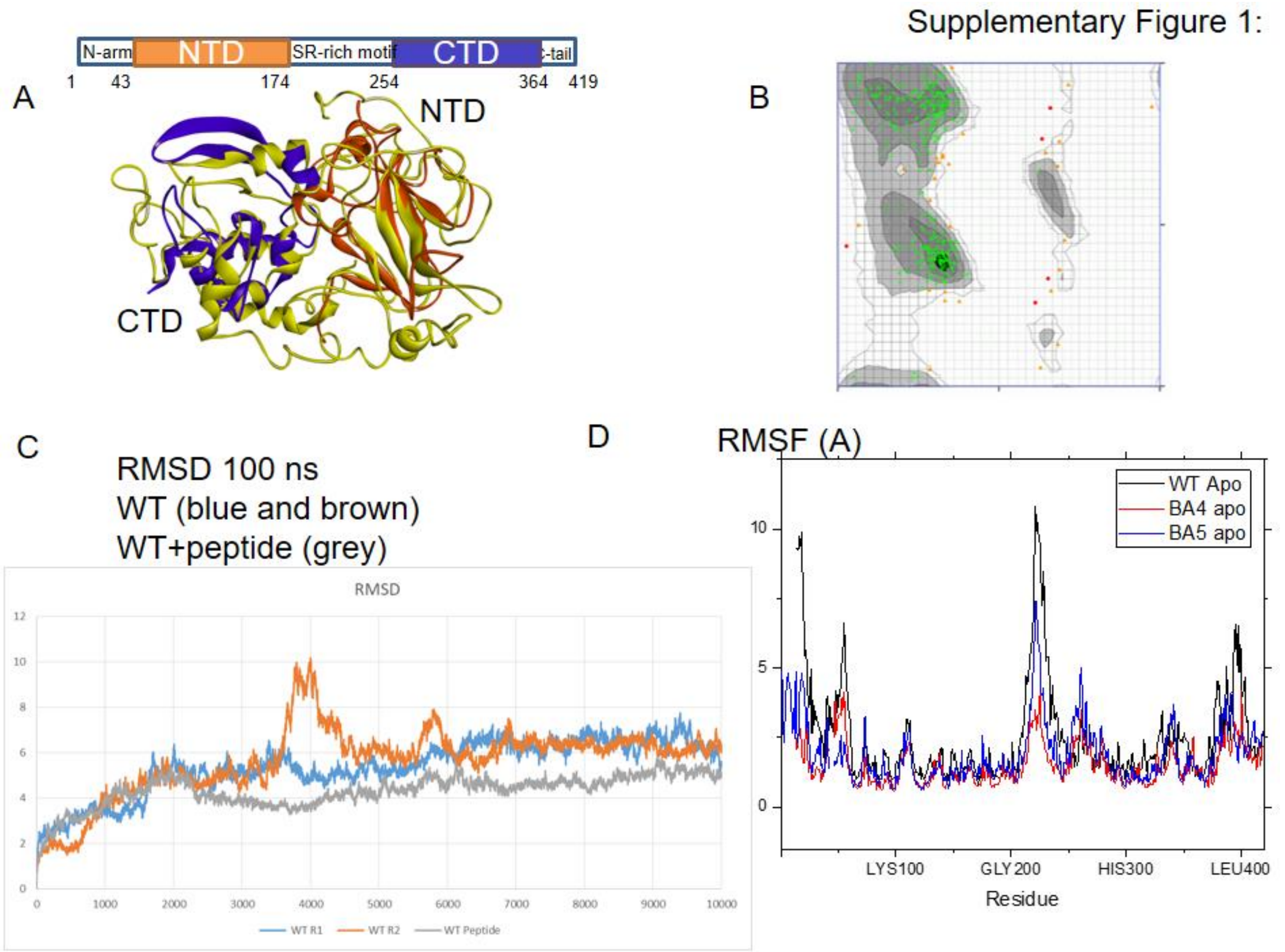

Supplementary Figure S2 : Melting curve of the N FL wt protein determined by DLS :
Zave correspond to the mean size of the protein in nm. Two transitions are seen, we attribute the first one at 43 °C to a transition from an oligomer (likely dimer) to a monomer and the second one around 54 °C to the monomer denaturation.

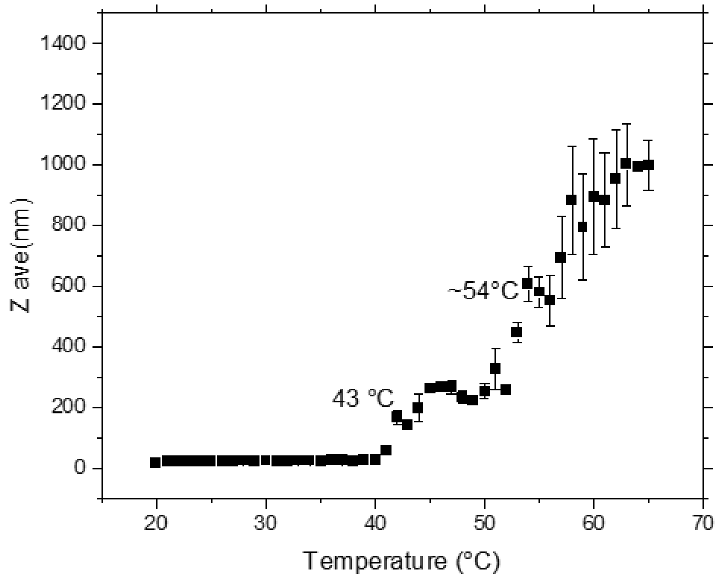

Supplement: Supplementary file 1 [file molecules-27-08094-s001.zip › molecules-1953977-supplementary.pdf]
